# Overexpression of ERCC3 is associated with poor prognosis in patients with pancreatic cancer

**DOI:** 10.7150/jca.54576

**Published:** 2021-03-05

**Authors:** Shujie Wang, Wenjing Liu, Yueli Ni, Lifeng Wang, Yuzhi Zhu, Qiong Shi, Zihan Yi, Wenjie Wang, Lili Liu, Lijuan Yang, Yingmin Kuang, Yuechun Zhu, Qiao Zhang, Zhe Yang

**Affiliations:** 1 Department of pathology, The First Affiliated Hospital of Kunming Medical University, Yunnan, China.; 2 Department of Biochemistry and Molecular Biology, School of Basic Medical Sciences, Kunming Medical University, Yunnan, China.; 3 Department of Clinical Laboratory, The Third Affiliated Hospital of Kunming Medical University (Tumor Hospital of Yunnan Province), Yunnan, China.; 4 Department of Medical Oncology, The Third Affiliated Hospital of Kunming Medical University (Tumor Hospital of Yunnan Province), Yunnan, China.; 5 Department of Organ Transplantation, The First Affiliated Hospital of Kunming Medical University, Yunnan, China.

**Keywords:** ERCC3, pancreatic cancer, prognosis biomarker, TCGA data mining, immunohistochemistry

## Abstract

Pancreatic cancer is associated with poor prognosis due to limited therapeutic options. Excision repair cross-complementing 3 (ERCC3) is an important member of nucleotide excision repair (NER) that is overexpressed in some cancers and may be regarded as a poor prognostic factor. Yet, its role in pancreatic cancer remains unclear. This study aimed to investigate the expression and functions of ERCC3 in pancreatic cancer patients and its relation with clinicopathological features. Our data suggested that the protein expression level of ERCC3 was higher in tumor tissues than in adjacent tissues. In addition, the expression of ERCC3 has shown to be associated with the tumor extent (*p*=0.035). Besides, analysis of the dataset in The Cancer Genome Atlas (TCGA) revealed that high expression of ERCC3 was associated with poor overall survival in pancreatic cancer patients (*p*=0.0136). In Cox regression analysis, ERCC3 was an independent prognostic factor for overall survival in pancreatic cancer (*p*<0.001). Furthermore, our *in vitro* data further suggested that the overexpression of ERCC3 significantly promoted pancreatic cancer (BxPC-3, CFPAC-1, and PANC-1 cells) proliferation, invasion, and migration. Taken together, this study suggested that high expression of ERCC3 might be a poor prognostic factor in human pancreatic cancer and might be used as a promising therapeutic target for pancreatic cancer treatment.

## Introduction

Pancreatic cancer is the 7^th^ leading cause of cancer-related deaths worldwide 1, with the highest incidence-to-morality ratio among all solid tumors 2. It has been estimated that there will be 57,600 new pancreatic cancer cases and 47,050 deaths in the United States in 2020 3. Moreover, with the overall 5-year relative survival rate of only 9% 4 and the continuous increase of the incidence and mortality rates, this type of cancer will become the second leading cause of cancer-associated death by the end of 2020 5.

Pancreatic ductal adenocarcinoma (PDAC) constitutes approximately more than 85% of all pancreatic cancer cases 1, among which only 20% of cases are eligible for surgery 6. The relapse rate is 80%, while the major cause of high incidence and death is a high metastatic rate 7. Therefore, novel diagnostic and prognostic biomarkers for pancreatic cancer are urgently required.

Excision repair cross-complementing 3 (ERCC3) is a 3'-5' DNA helicase, the largest subunit of transcription factor IIH (TFIIH) 8. It is also known as XPB. ERCC3 is regarded as an important member of nucleotide excision repair (NER) 8. It participates in human DNA-repair disorders xeroderma pigmentosum (XP), Cockayne's syndrome (CS), and trichothiodystrophy (TTD) 8, 9. Recent evidence revealed elevated ERCC3 expression in epithelial ovarian cancer and hepatocellular carcinoma 10, 11. Moreover, the genetic variants in ERCC3 may contribute to the development of breast cancer 12, lung cancer 13, osteosarcoma 14, and bladder cancer 15. In addition, testing of triptolide in conditionally reprogrammed patient-derived carcinoma revealed ERCC3-MYC interactions and variable sensitivity between lines 16. Yet, so far, no studies have examined the expression profile of ERCC3 in human pancreatic cancer, and the clinical significance of ERCC3 in human pancreatic cancer is still unknown. In this study, the dataset of ERCC3 mRNA expression from The Cancer Genome Atlas (TCGA) was used to analyze the role of ERCC3 in pancreatic cancer and its clinicopathological significance.

## Materials and Methods

### Tissue microarray

The tissue microarray (HPan-Ade120Sur-01), including 63 human pancreatic cancer tissues with 57 relevant normal adjacent tissues (6 pancreatic cancer tissues without matched adjacent tissues), was purchased from Shanghai Outdo Biotech Co., Ltd. Immunohistochemistry (IHC) analyzed the protein expression levels. The detailed characteristics of patients who were staged according to the 7^th^ classification Staging Manual of the American Joint Commission on Cancer (AJCC) are listed in **Table 1**.

### Immunohistochemistry

The immunohistochemical assay was performed as previously described 17 using a rabbit monoclonal anti-XPB antibody (1:2000, Abcam, #ab99322). Subsequently, all specimens were stained by using Envision Detection Kit (GK500705, DAKO A/S) and counterstained with hematoxylin (ZLI‑9615, ZSGB-BIO).

### Immunohistologic analysis

Brownish-yellow for nuclear staining was regarded as a positive protein expression. The expression level of ERCC3 was determined by staining intensity and proportions of positive cells according to the Remmele-score system by two independent pathologists in a blinded fashion. Microscopically, six random fields of vision near the core of each tumor were selected. The number of positive cells in each selected field was counted using a 20× magnification, and then the average count was calculated. The staining intensity was rated as 1 (weak intensity), 2 (moderate intensity), 3 (strong intensity), and the proportion of positive cells in each field was regarded as follows: 0 (0~5%), 1 (6%~25%), 2 (26%~50%), 3 (51%~75%) and 4 (76%~100%). The final scores of ERCC3 expression levels were based on the multiplication of staining intensity and proportions of positive cells and defined as follows: negative (-), 0 points; weak (+), 1~4 points; moderate (++), 5~8 points; and strong (+++), 9~12 points. The final scores in negative and weak groups were considered as low expression, while the final scores in moderate and strong groups were classified as high expression.

### TCGA pancreatic cancer data mining

The data of 177 pancreatic cancer patients with ERCC3 mRNA expression information were acquired from TCGA. All clinical information, such as survival status, histologic grade, and time to follow-up for each patient was also accessible from TCGA.

### Cell culture and establishment of stable cell lines

Three human pancreatic cancer cell lines (BxPC-3, CFPAC-1, and PANC-1) were purchased from Kunming Cell Bank, Chinese Academy of Sciences. All these cell lines were cultured in Dulbecco's modified Eagle's medium (DMEM, BI) supplemented with 10% fetal bovine serum (FBS, BI) at 37 °C with a 5% CO_2_ water-saturated environment.

To establish cell lines with ERCC3 stable overexpression, BxPC-3, CFPAC-1, and PANC-1 cells were transfected with 2 μl lentivirus carrying an overexpression of ERCC3, at a density of 1×10^5^/ml in 6 well plates; cells transfected with 2 μl lentivirus without overexpression of ERCC3 were used as controls. Puromycin selection (5 mg/ml) was performed after 72 h of transfection. The culture medium containing puromycin was changed every two days. When more than 95% of cells performed green fluorescence signals, real-time RT-PCR and Western blot were executed to identify the stable cells. Established stable cell lines were collected after about 21 days of culturing and screening.

### Western blot

Pancreatic cancer cells were lysed on ice using RIPA lysis buffer (Solarbio, #R0010) with protease inhibitors (Solarbio, #P0100). The protein concentrations were determined using a BCA^TM^ Protein Assay Kit (Applygen). Equal amounts of protein were separated by 10% sodium dodecyl sulfate-polyacrylamide gel electrophoresis (SDS-PAGE) and then transferred to polyvinylidene fluoride (PVDF) membranes (Millipore, #IPVH00010). The membranes were blocked by 1×TBST buffer containing 5% skim milk for 2 h and later incubated with primary antibodies overnight at 4 °C. After washing the membranes three times in 1×TBST on a shaker, the detection was performed by the appropriate secondary antibodies for 2 h at room temperature. Finally, Blots were detected using an enhanced chemiluminescence reagent ECL kit (Advansia, #K-12045-D50).

Antibodies used in this study were listed as follow: anti-XPB antibody (1:2000, Abcam, #ab190698), anti-Bcl_2_ antibody (1:1000, Proteinch, #12789-1-AP), anti-Bax antibody (1:1000, Proteinch, #50599-2-Ig), anti-caspase 3 (1:500, CST, #9662), anti-MMP9 (1:1000, Abcam, #ab76003), anti-MMP13 (1:3000, Abcam, #ab39012), anti-cyclin E1 antibody (1:2000, Proteinch, #11554-1-AP), anti-CDK 2 antibody (1:1000, Proteinch, #10122-1-AP), anti-cyclin D1 antibody (1:800, Proteinch, #60186-1-Ig), anti-CDK 4 antibody (1:500, Proteinch, #11026-1-AP), anti-Ki-67 antibody (1:500, Abcam, #ab15580), anti-GAPDH antibody (1:2000, Bioss, # bs-2188R).

### Real-time RT-PCR

Total RNA was extracted using the Trizol Reagent (TakaRa, #9109) and reversed transcription into cDNA using Thermo RT Kit (Thermo, #K1622). DNA Green Master (ROX) (Roche, #04913914001) was used for real-time PCR according to the manufacturer's protocol. Primers used in this experiment were listed as follows: ERCC3: F: 5'-ATGGGCAAAAGAGACCGAGC-3', R: 5'TCGTCCTTCAGCGGCATTT-3'; U6: F: 5'- CTCGCTTCGGCAGCACA -3', R: 5'- AACGCTTCACGAATTTGCGT-3'.

### Cell proliferation assay

Cell proliferation was analyzed using the CCK-8 assay. Pancreatic cancer cells were plated at a density of 1×10^4^/ml in 96 well plates and treated differently for 0 h, 24 h, 48 h, and 72 h. After each time point, the cells were incubated with 10 μl CCK-8 (APEBIO, # k1018) at 37 °C for 2 h in dark. The fluorescence intensity was measured by a microplate reader (Thermo Scientific, #51119200) at spectrometric absorbance of 450 nm.

### Cell apoptosis assay

Cell apoptosis was analyzed using an Annexin V-633/ Propidium Iodide (PI) apoptosis detection kit (DOjinDO, #AD11) based on the manufacturer's protocol 18. After ten hours' hunger, that was all the cells were cultured in DMEM supplemented with no FBS. Then cells were collected, washed with phosphate-buffered saline (PBS), and resuspended in 1×Annexin V binding solution at a final concentration of 1×10^6^ cells/ml. 5 µl Annexin V-633 binding buffer and 5 µl PI solution were added to 100 µl of the prepared cell suspension. After 15 min at room temperature in dark, 200 µl binding buffer were added. Finally, the cells were detected by PARTEC CyFlow Space flow cytometry.

### Transwell assay

The invasion and migration assays were performed using Transwell chambers with 8 μm core filters (Corning, #3422). There was pre-diluted Matrigel (diluted at a ratio of 1:3 with serum-free DMEM medium) (BD, #356234) in filters for invasion assays, while no matrigel was used for the migration assays. Briefly, 2×10^4^ cells/100 μl with serum-free DMEM was added to the upper chamber, while 600 μl complete medium was added to the lower chamber. After 36 h, cells on the bottom surface were fixed with 4% paraformaldehyde for 15 min, washed by PBS, stained by 0.1% crystal violet at 37 °C for 30 min and observed under a digital microscope (Leica).

### Statistical analysis

Statistical analyses were performed by SPSS 20.0 (SPSS, Chicago, IL). For immunohistologic analysis, the correlation between ERCC3 expression and clinicopathological features of each patient were performed using Fisher exact test. For TCGA data, based on the median levels of ERCC3 mRNA expression, patients were separated into two groups-high ERCC3 and low ERCC3. For Kaplan-Meier curves, the Log-rank test was used to measure the statistical difference between the two groups. Using a Cox proportional hazards model, variables with *p*<0.05 at the univariate analysis were included in multivariate survival analysis. A *p*<0.05 was considered to be statistically significant.

## Results

### ERCC3 expression in clinical-pathological specimens

**Figure 1:**
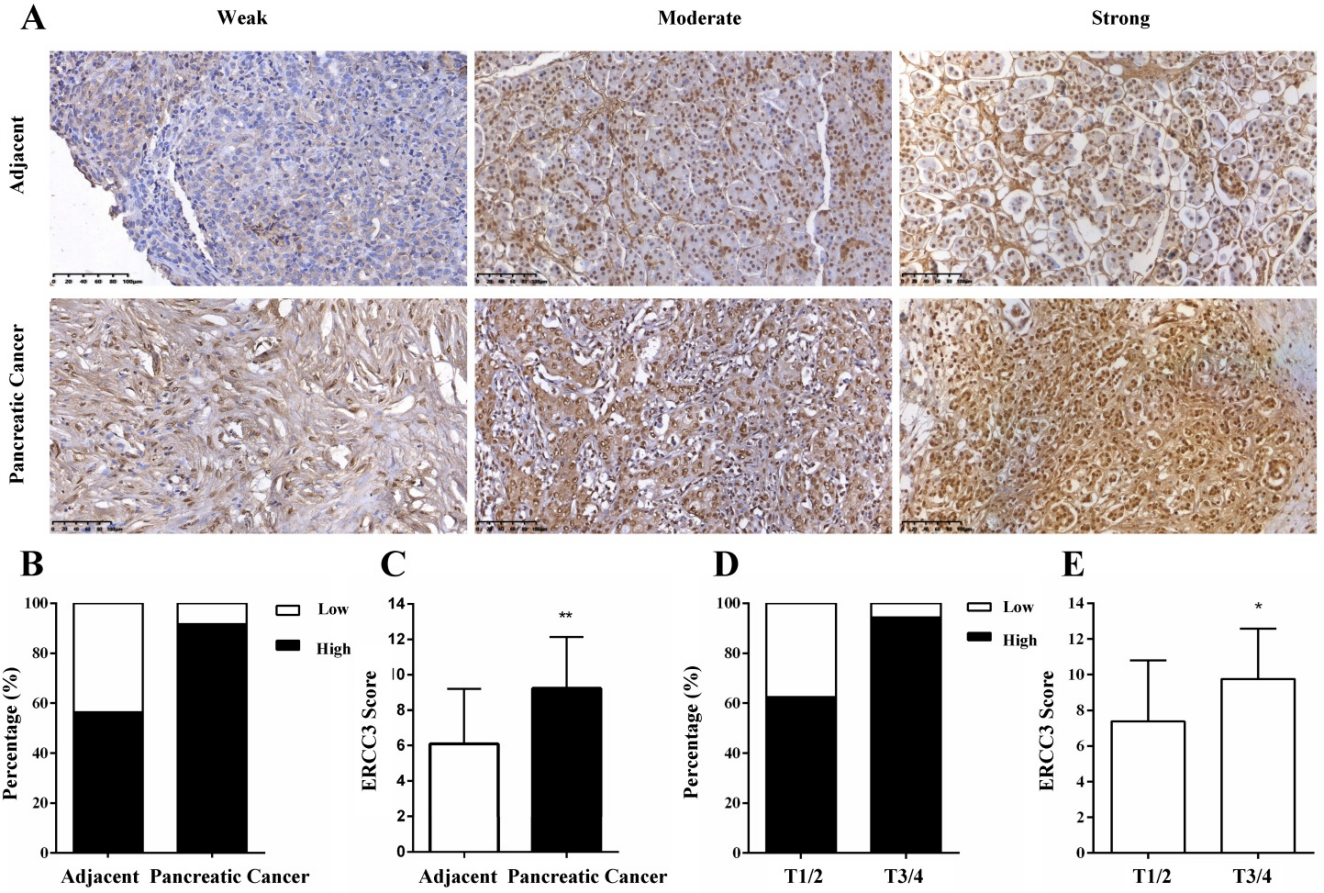
** Representative images and statistical analysis of immunohistochemical staining for ERCC3 in pancreatic cancer and adjacent non-tumor pancreas tissues. (A)** IHC analysis of ERCC3 expression in paired adjacent normal tissues (top panel) and pancreatic cancer tissue specimens (bottom panel) showed weak, moderate, and strong ERCC3 staining. All images were captured by a 20× objective lens. Scale bar: 100 µm. **(B)** Percentage of pancreatic cancer samples and matched normal tissues with low and high expression of ERCC3. **(C)** Statistical analysis of ERCC3 expression in pancreatic cancer and matched adjacent normal tissues by *χ*2 test based on IHC staining scores (**p<0.01, vs. matched adjacent normal tissues). **(D)** The percentage of pancreatic cancer patients in T1/2 and T3/4 stage, with low and high expression levels of ERCC3. **(E)** Statistical analysis of ERCC3 expression in T1/2 and T3/4 based on IHC scores analyzed with Fisher exact test (*p<0.05).

The expression of ERCC3 in pancreatic cancer specimens and relevant cancer-adjacent normal pancreatic tissues were first investigated by IHC and evaluated by staining scores. After IHC, there were 3 pancreatic cancer and 2 adjacent cases tissues were not available. The results showed that ERCC3 was predominantly located in the nucleus of the pancreas cells (**Figure 1A**). In addition, weak, moderate and strong positive expression levels of ERCC3 were detected in 8.3% (5/60), 30% (18/60), and 61.7% (37/60) of the pancreatic cancer tissues (**Figure 1A**, bottom panel), and in 43.6% (24/55), 41.8% (23/55) and 14.5% (8/55) of the noncancerous pancreas tissues (**Figure 1A,** top panel), respectively. Most importantly, 91.7% (55/60) of pancreatic cancer specimens and 56.3% (31/55) of adjacent samples had high expression of ERCC3 (**Figure 1B**), and the staining score of ERCC3 in pancreatic cancer and associated-adjacent tissues was significantly different (*p*<0.05, *χ*^2^ test, **Figure 1C**). These results suggested that compared with normal adjacent tissues, ERCC3 expression was markedly increased in pancreatic cancer tissues.

### Correlation between ERCC3 expression and clinicopathological features

To explore the role of ERCC3 in pancreatic cancer, the correlations between ERCC3 expression levels and the clinicopathological parameters of the 63 pancreatic cancer patients (**Table 1**) were analyzed (3 pancreatic cancer tissues were not available after IHC). The results suggested that the expression of ERCC3 was associated with the tumor extent (*p*=0.035, Fisher exact test) instead of other clinicopathological features (**Table 2**). Furthermore, the data showed that 62.5% (5/8) T stage 1 and 2 (T1/2) cancer and 94.4% (34/36) T3/4 cases exhibited strong immunopositivity (**Figure 1D**). Compared to T1/2 tissues, the final score of ERCC3 was remarkably increased in the T3/4 stage (*p*<0.05, Fisher exact test, **Figure 1E**). These data suggested that ERCC3 expression is clinically relevant.

### Expression profile and prognostic value of ERCC3 by TCGA data mining

**Figure 2:**
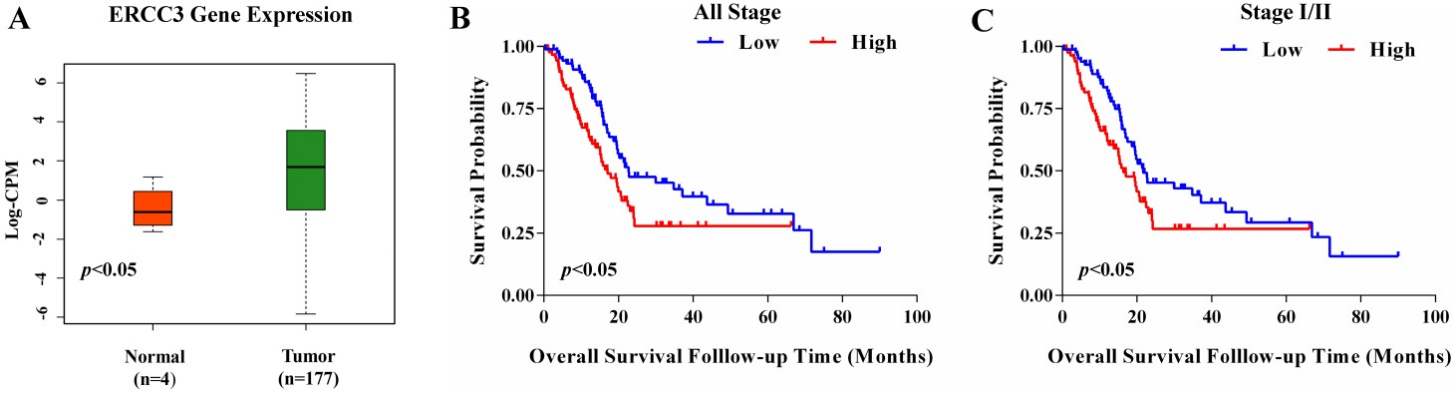
** The expression profile and prognostic significance of ERCC3 in pancreatic cancer based on TCGA data mining.** Four normal pancreas tissues and 177 pancreatic cancer cases were downloaded from TCGA. According to the median of ERCC3 mRNA expression, pancreatic cancer patients were divided into the ERCC3 high expression group (n=89) and the ERCC3 low expression group (n=88). **(A)** Boxplot showed ERCC3 gene expression levels in pancreatic cancer specimens compared with normal pancreas tissues using the Mann-Whitney U test (*p*<0.05). Kaplan-Meier overall survival curves for all 177 patients with all stage (**B,** *p*<0.05), 166 patients with stage I/II pancreatic cancer (**C,** *p*<0.05). Significance measures were conducted using log-rank test (vs. ERCC3 low group).

Next, we investigated the ERCC3 mRNA expression and clinical information of 177 pancreatic cancer cases extracted from TCGA. ERCC3 gene expression level was higher in pancreatic cancer specimens compared with normal pancreas tissues (*p*<0.05, **Figure 2A**). Moreover, significant differences in ERCC3 were observed among different pathological grades (*p*<0.001, **Table 3**). Therefore, these findings encouraged us to identify whether ERCC3 mRNA expression profile was of significance in this current cohort of pancreatic cancer. 177 pancreatic cancer cases were assigned into the ERCC3-low group (n=88) and ERCC3-high group (n=89) according to the median value of ERCC3 mRNA expression levels as a cut-off. Based on the different groups, time to the last follow-up was plotted in the Kaplan-Meier curve. These results suggested that pancreatic cancer patients with all stage (**Figure 2B,** *p*=0.0136) and Ⅰ/Ⅱ stage (**Figure 2C,** *p*=0.0257) in the ERCC3 high group had a shorter survival rate. However, 8 pancreatic cancer patients with stage III/IV showed that the ERCC3 expression was not associated with prognosis (**Figure S1**). Furthermore, Cox regression was performed to analyze the dataset, and the results showed that ERCC3 could be considered as an independent prognostic factor for overall survival in pancreatic cancer, (*p*<0.001, **Table 4**). Overall, all these data indicated that ERCC3 overexpression (OE) might present a poor survival biomarker for pancreatic cancer patients.

### The functional role of ERCC3 in pancreatic cancer cells *in vitro*

**Figure 3:**
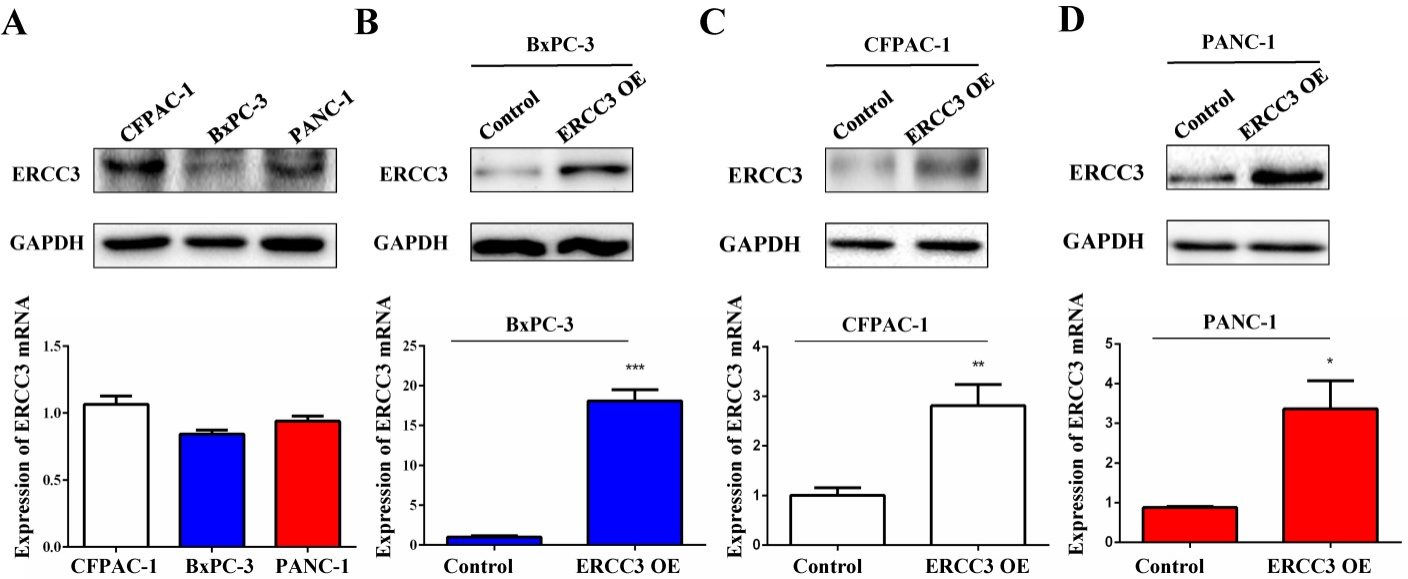
** The constitutive expression and transfection efficiency of ERCC3 in BxPC-3, CFPAC-1, and PANC-1 cells detected by Western blot and real-time RT-PCR.** ERCC3 protein and mRNA expression levels in wild type pancreatic cancer cells** (A)**, stable transfected BxPC-3 **(B)**, CFPAC-1 **(C)** and PANC-1 **(D)** cells with ERCC3 overexpression and the control cells were detected by Western blot and real-time RT-PCR. Statistical analysis was conducted by using an unpaired *t*-test (B-D) (*p<0.05, **p<0.01, *** *p*<0.001, vs. each control).

**Figure 4:**
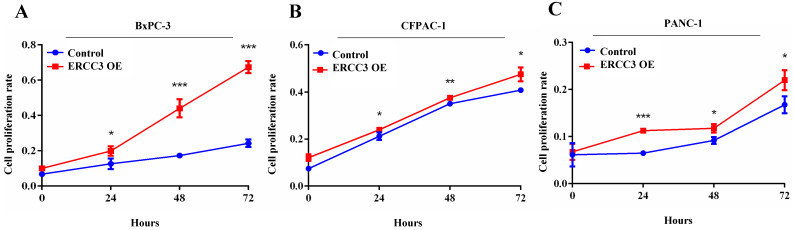
** The effect of ERCC3 on pancreatic cancer cell proliferation.** The proliferation rates of BxPC-3 **(A)**, CFPAC-1 **(B)**, and PANC-1 **(C)** cells with ERCC3 overexpression and the control cells were detected by CCK-8 assay. Statistical analysis was conducted by using an unpaired *t*-test (A-C) (*p<0.05, **p<0.01, *** *p*<0.001, vs. each control).

**Figure 5:**
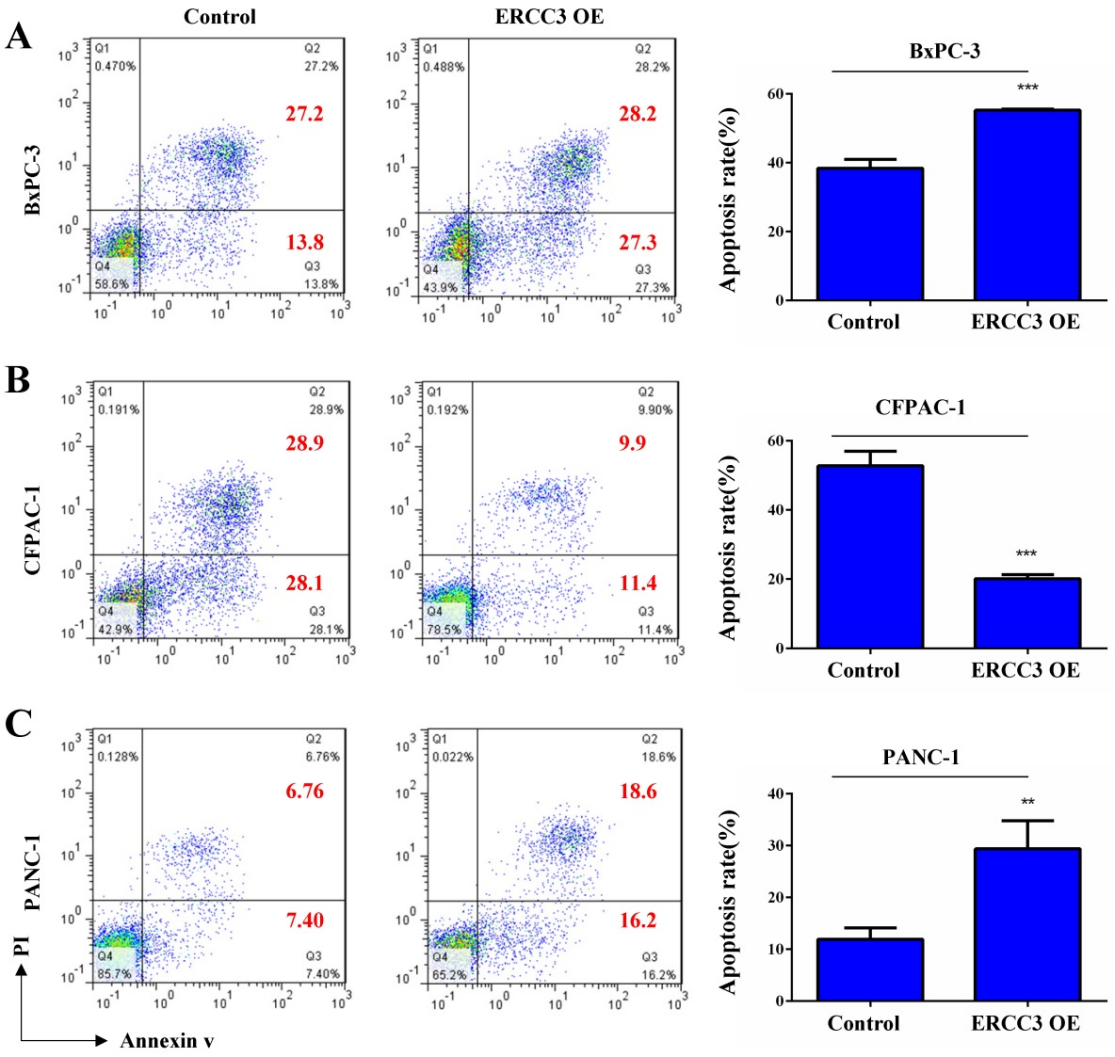
** The effect of ERCC3 on pancreatic cancer cell apoptosis.** The apoptosis rates of BxPC-3 **(A)**, CFPAC-1 **(B)**, and PANC-1 **(C)** cells with ERCC3 overexpression and the control cells were detected by flow cytometry analysis (cells were stained with Annexin V-633/PI). Statistical analysis was counted by using an unpaired *t*-test (A-C) (**p<0.01, *** *p*<0.001, vs. each control).

**Figure 6:**
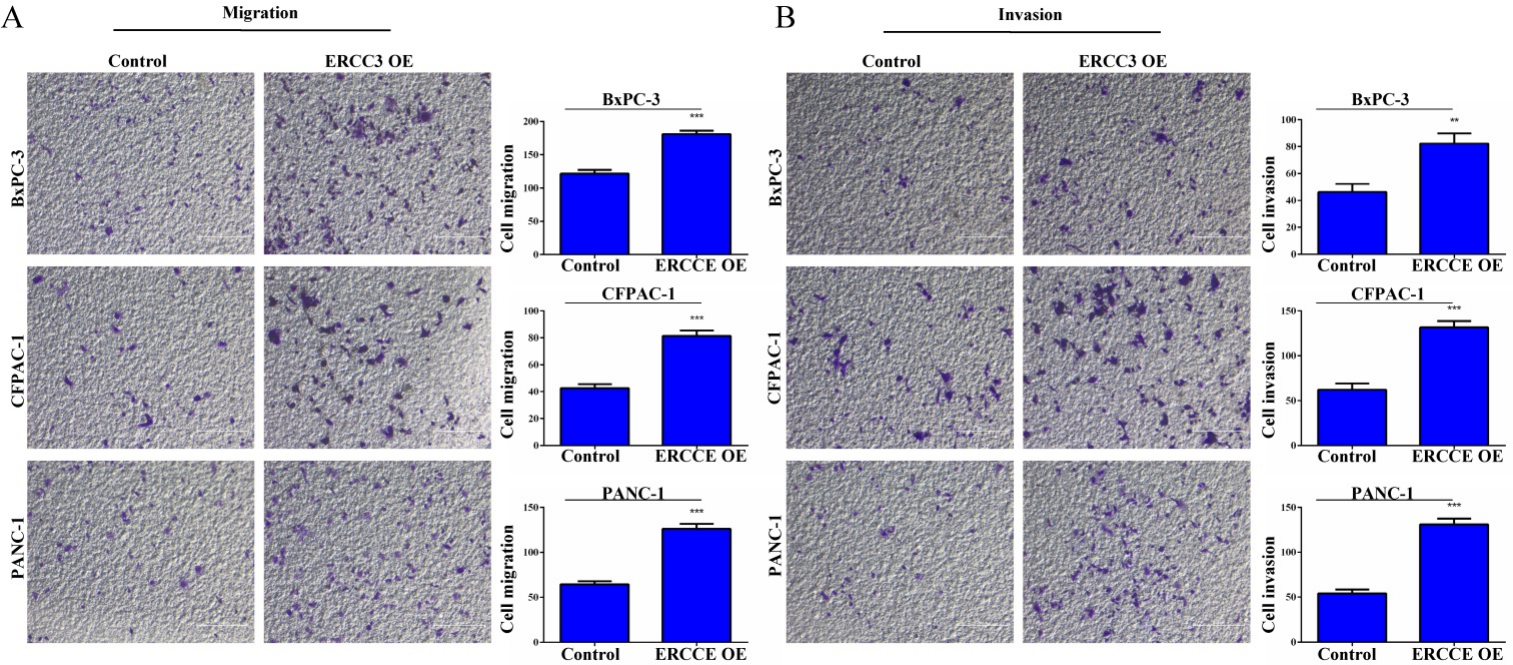
** The effect of ERCC3 on pancreatic cancer cell migration and invasion. (A)** The effect of ERCC3 on cell migration capabilities in BxPC-3, CFPAC-1, and PANC-1 cells was detected by transwell assay. Scale bar: 200 µm. **(B)** The effect of ERCC3 on cell invasion potential in BxPC-3, CFPAC-1, and PANC-1 cells was observed by matrigel-transwell assay. Scale bar: 200 µm (**p<0.01, *** *p*<0.001, vs. each control).

To determine the function of ERCC3 in pancreatic cancer, BxPC-3, CFPAC-1, and PANC-1 cells were transfected with lentivirus vectors (**Figure 3**). The CCK-8 assay was used to explore the effect of ERCC3 on tumor cell growth. As shown in **Figure 4**, ERCC3 OE promoted BxPC-3, CFPAC-1, and PANC-1 cells proliferation compared with control cells (*p*<0.05, unpaired *t*-test). Furthermore, the apoptosis assay suggested that ERCC3 OE significantly increased the apoptosis rate of BxPC-3 and PANC-1 cells while decreased the rate of CFPAC-1 cells (**Figure 5**). Additionally, a transwell assay was performed to evaluate the impact of ERCC3 expression on migration and invasion in pancreatic cancer cells. Compared with the control groups, ERCC3 OE led significantly increased cell migration (**Figure 6A**) and invasion (**Figure 6B**) of BxPC-3, CFPAC-1, and PANC-1 cells. These data implied that ERCC3 might serve as a tumor accelerator of pancreatic cancer cells, especially in proliferation and metastasis.

**Figure 7:**
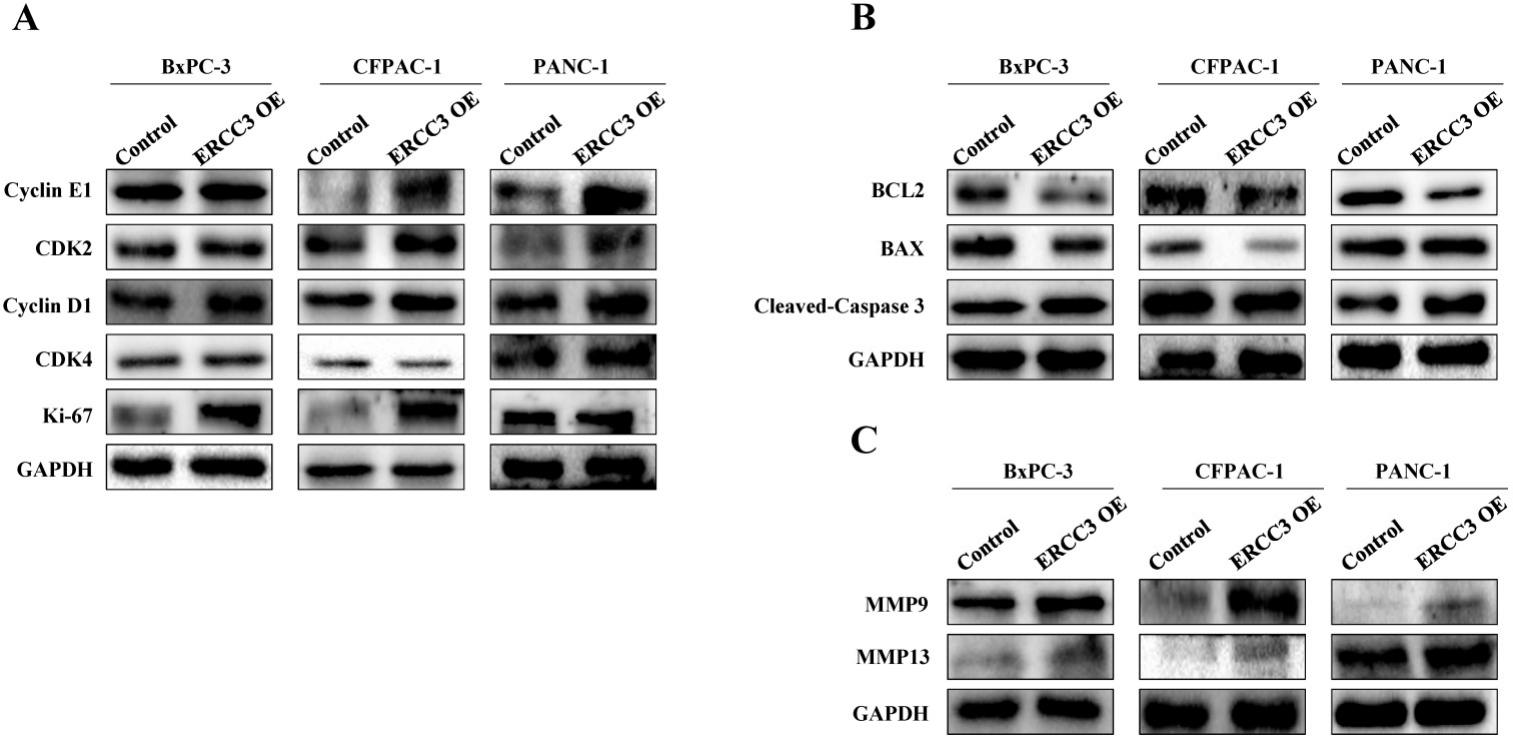
** The expression of factors related to proliferation, apoptosis, and metastasis in pancreatic cancer cells overexpressing ERCC3.** Western blot assays of the expression changes of proliferation **(A)**, apoptosis **(B)** and metastasis **(C)** related factors following ERCC3 overexpression in pancreatic cancer cells.

Western blot was next used to evaluate the expression of factors related to proliferation, apoptosis, and metastasis in pancreatic cancer cells (**Figure 7**). Compared with the control group, the ERCC3 OE group promoted proliferation-associated gene expression (**Figure 7A**). The anti-apoptotic gene Bcl-2 was down-regulated in BxPC-3, CFPAC-1, and PANC-1 ERCC3 OE cells (**Figure 7B**). In addition, the pro-apoptotic gene Bax did not significantly change in BxPC-3 and PANC-1 ERCC3 OE cells, but was down-regulated in CFPAC-1 ERCC3 OE cells (**Figure 7B**). Similarly, the pro-apoptotic gene cleaved-caspase 3 was up-regulated in both BxPC-3 and PANC-1 ERCC3 OE cells (**Figure 7A**), but was down-regulated in CFPAC-1 ERCC3 OE cells. Besides, ERCC3 OE groups promoted the expression of both matrix metalloproteinase 9 (MMP9) and matrix metalloproteinase 13 (MMP13) (**Figure 7B**).

In brief, the above results indicated that ERCC3 OE could promote proliferation, migration and invasion of BxPC-3, CFPAC-1, and PANC-1 cells. It also increased the apoptosis rate of BxPC-3 and PANC-1 cells while decreased the rate of CFPAC-1 cells. Besides, ERCC3 expression was increased in pancreatic cancer tissues and it was clinically relevant. What's more, ERCC3 OE was associated with poor prognosis for pancreatic cancer patients.

## Discussion

Pancreatic cancer usually has a poor prognosis due to high relapse rate and metastasis after surgical resection. Surgical resection is the only curative treatment, and the majority of patients are diagnosed at an advanced stage. Thus, there is an urgent need to develop sensitive and specific biomarkers for diagnosis.

Recent studies suggested ERCC3 overexpression as a potential prognostic factor in a patient with hepatocellular carcinoma 11. In this study, we examined the expression profile of ERCC3 in pancreatic cancer and its clinical significance. A series of experiments were performed to confirm whether ERCC3 could be used as a promising biomarker for improving the diagnosis and predicting prognosis in patients with pancreatic cancer. Our immunohistochemical analysis showed that ERCC3 expression was notably increased in pancreatic cancer tissues and clinically relevant. Furthermore, from the TCGA dataset, we found that high expression of ERCC3 was associated with poor overall survival in pancreatic cancer patients. It acted as a powerful independent predictor of the prognosis for pancreatic cancer patients. This study first demonstrated the correlation between ERCC3 expression and prognosis of pancreatic cancer patients.

Next, we examined the exact mechanism through which ERCC3 affected prognosis and outcomes in pancreatic cancer patients. Our *in vitro* results showed that ERCC3 had a noticeable effect in promoting pancreatic cancer cells proliferation. In addition, overexpression of ERCC3-further promoted cell migration and invasion. This data was further confirmed by Western blot; higher expression levels of ERCC3 up-regulated the expression of MMP9 and MMP13. Previous studies suggested that MMPs were contacted with the epithelial to mesenchymal transition (EMT), which was considered as a hallmark of cancer progression to metastasis 19. In addition, a previous study confirmed the pro-angiogenic role of MMPs, which were also related to the occurrence and development of cancers 20, 21.

Furthermore, we discovered that ERCC3 could affect pancreatic cancer cell apoptosis. Overexpression of ERCC3 significantly increased the apoptosis rate of BxPC-3 and PANC-1 cells, but not in CFPAC-1 cells. A possible reason for this might be the different sources of those cell lines: although all three cell lines were pancreatic cancer cell lines, only CFPAC-1 cell lines derived from liver metastasis. As was reported in previous study, microtubule associated protein 9 (MAP9) inhibited liver tumorigenesis by suppressing ERCC3, and ERCC3 promoted LO2 and HepG2 cell lines proliferation and invasion 11. Therefore, we supposed that ERCC3 overexpression might inhibit pancreatic cancer cells apoptosis when distant metastasis occurred. Yet, this data needs to be examined in future studies. Another possible reason for the controversial result of apoptosis rate in these three cell lines might be the heterogeneous nature of cancer. As evidence, there were two human prostate cancer cell lines (MDA PCa 2a and MDA PCa 2b) derived from different areas of the same tumor specimen, but they had different genetic features and different phenotypes, such as morphology and growth 22. Similarly, although K562, KCL22, and LAMA84 were Ph^+^ chronic myelogenous leukemia (CML) cell lines and represented the same pathological phenotype, K562 and KCL22 cells preferentially expressed proteins associated in drug resistance, while LAMA84 cells preferentially expressed proteins involved with invasion 23. Besides, a study showed that bone morphogenetic protein 4 (BMP4) could promote HCC1954, MDA231 and MDA-361 these three breast cancer cells migration but showed reverse effect on T-47D breast cancer cells. The different phenotypes response in cell migration assay after the stimulation of BMP4 could be explained by different signaling cascades in downstream 24. As noted above, it is reasonable to suppose that the possibilities for the different results of apoptosis rate in Bxpc-3, PANC-1 and CFPAC-1 are the heterogeneous nature of cancer and different signaling cascades activated in different cells when ERCC3 is overexpressed in downstream. This hypothesis will be further investigated in future work.

To sum up, this study demonstrated that ERCC3 was overexpressed in pancreatic cancer. Moreover, we found that the high expression of ERCC3 could be considered as an independent unfavorable prognostic biomarker for pancreatic cancer patients. In addition, ERCC3 could promote proliferation, invasion, and migration of pancreatic cancer cells *in vitro*. Yet, detailed molecular mechanisms through which ERCC3 regulates the progression of pancreatic cancer need to be further elucidated.

## Supplementary Material

Supplementary figure.

## Figures and Tables

**Table 1:** List of 63 pancreatic cancer tissues

Characteristics	n (%)
**Gender**	
Male	36 (57.1%)
Female	27 (42.9%)
**Age at surgery**	
<60	21 (33.3%)
≥60	42 (66.7%)
**Tumor extent^a^**	
T1	2 (3.2%)
T2	7 (11.1%)
T3	38 (60.3%)
Not Available	16 (25.4%)
**Lymph node metastasis^a^**	
N0	35 (55.5%)
N1	25 (39.7%)
Not Available	3 (4.8%)
**Distant metastasis^a^**	
M0	59 (93.7%)
M1	4 (6.3%)
**Tumor max diameter (cm)**	
<4	23 (36.5%)
≥4	40 (63.5%)
**Histologic grade^b^**	
G1-2	37 (58.7%)
G3	26 (41.3%)
**TNM stage^a^**	
I/II	50 (79.4%)
III/IV	4 (6.3%)
Not Available	9 (14.3%)

^a^ According to the 7^th^ classification Staging Manual of the American Joint Commission on Cancer (AJCC); ^b^ Based on the World Health Organization Classification of Tumours 2000.

**Table 2:** Correlation between the expression of ERCC3 and clinicopathologic parameters in pancreatic cancer

Characteristics	Total	Expression of ERCC3		*p* value
		Low	High	
**Gender**				
Male	35	2	33	0.640^a^
Female	25	3	22	
**Age at surgery**				
<60	21	2	19	1.000^a^
≥60	39	3	36	
**Tumor extent**				
T1/2	8	3	5	**0.035^a^**
T3/4	36	2	34	
**Lymph node metastasis**				
N0	33	3	30	1.000^a^
N1	25	2	23	
**Distant metastasis**				
M0	56	4	52	0.301^a^
M1	4	1	3	
**Tumor max diameter (cm)**				
<4	21	2	19	1.000^a^
≥4	39	3	36	
**Histologic grade**				
G1-2	35	2	33	0.640^a^
G3	25	3	22	
**TNM stage**				
I/II	47	4	43	0.347^a^
III/IV	4	1	3	

Data was not available (NA) for some cases: Tumor extent (NA = 16), Lymph node metastasis (NA = 2), TNM stage (NA = 9); ^a^, Fisher exact test; Significant *p*-value is in **bold.**

**Table 3:** Correlation between the expression of ERCC3 and clinicopathologic parameters in pancreatic cancer analyzed by TCGA data mining

Characteristics	Expression of ERCC3		*p* value
	No. (%)	avg. value	
**Sex**			
Male	97 (54.8%)	1.181	0.893^a^
Female	80 (45.2%)	1.216	
**Age at surgery**			
<60	57 (32.2%)	1.716	0.056^a^
≥60	120 (67.8%)	0.950	
**T stage**			
T1/2	28 (15.8%)	-0.190	0.088^b^
T3/4	147 (83.1%)	1.538	
Tx	1 (0.6%)	-5.300	
Not Available	1 (0.6%)	-3.668	
**N stage**			
N0	48 (27.1%)	0.680	0.261^b^
N1	123 (69.5%)	1.547	
Nx	5 (2.8%)	-1.173	
Not Available	1 (0.6%)	-5.170	
**M stage**			
M0	78 (44.1%)	1.755	0.083^b^
M1	5 (2.8%)	-0.497	
Mx	94 (53.1%)	0.824	
**Pathological grade**			
G1	28 (15.8%)	-1.370	**<0.001^b^**
G2	96 (54.2%)	1.568	
G3	49 (27.7%)	2.241	
G4	2 (1.1%)	-1.051	
Gx	2 (1.1%)	-4.010	
**TNM stage**			
I/II	166 (93.8%)	1.278	0.365^b^
III/IV	8 (4.5%)	0.562	
Not Available	3 (1.7%)	-1.584	

^a^, Mann-Whitney U test; ^b^, Cruskal-Wallis H (K) test. Significant *p*-value was in **bold.**

**Table 4:** Univariate and multivariate Cox regression analysis of the association of ERCC3 expression and other clinicopathologic features with overall survival in pancreatic cancer analyzed by TCGA data mining

Characteristics	Univariate			Multivariate		
	HR	95% CI	*p* value	HR	95% CI	*p* value
Sex	0.817	0.545-1.226	0.330			
Age at surgery	1.028	1.007-1.050	**0.008**	1.030	1.010-1.050	**0.003**
T stage	1.378	0.854-2.222	0.189			
N stage	1.463	1.004-2.132	**0.048**	1.858	1.173-2.941	**0.008**
M stage	0.923	0.749-1.136	0.449			
Pathological grade	1.409	0.955-2.078	0.084			
TNM stage	0.422	0.158-1.128	0.086			
ERCC3 expression	1.175	1.092-1.265	**<0.001**	1.193	1.105-1.288	**<0.001**

Abbreviations: HR, hazard ratio; CI, confidence interval. Significant *p*-value was in **bold.**

## Abbreviations

ERCC3: excision repair cross-complementing 3
NER: nucleotide excision repair
TCGA: The Cancer Genome Atlas
PDAC: pancreatic ductal adenocarcinoma
TFIIH: transcription factor IIH
XP: xeroderma pigmentosum
CS: Cockayne's syndrome
TTD: trichothiodystrophy
AJCC: American Joint Commission on Cancer
IHC: immunohistochemistry
DMEM: Dulbecco's modified Eagle's medium
FBS: fetal bovine serum
SDS-PAGE: sodium dodecyl sulfate-polyacrylamide gel electrophoresis
PVDF: polyvinylidene fluoride
PI: propidium iodide
PBS: phosphate-buffered saline
OE: overexpression
MMP9: matrix metalloproteinase 9
MMP13: matrix metalloproteinase 13
EMT: epithelial to mesenchymal transition
MAP9: microtubule associated protein 9
CML: chronic myelogenous leukemia
BMP4: bone morphogenetic protein 4
