# Supplementary material for: Overexpression of ERCC3 is associated with poor prognosis in patients with pancreatic cancer
Source: J Cancer. 2021 Mar 5;12(9):2550–9. doi: 10.7150/jca.54576 (PMC8040713; doi:10.7150/jca.54576)
Supplement: Supplementary file 1 — Supplementary figure. [file jcav12p2550s1.pdf]

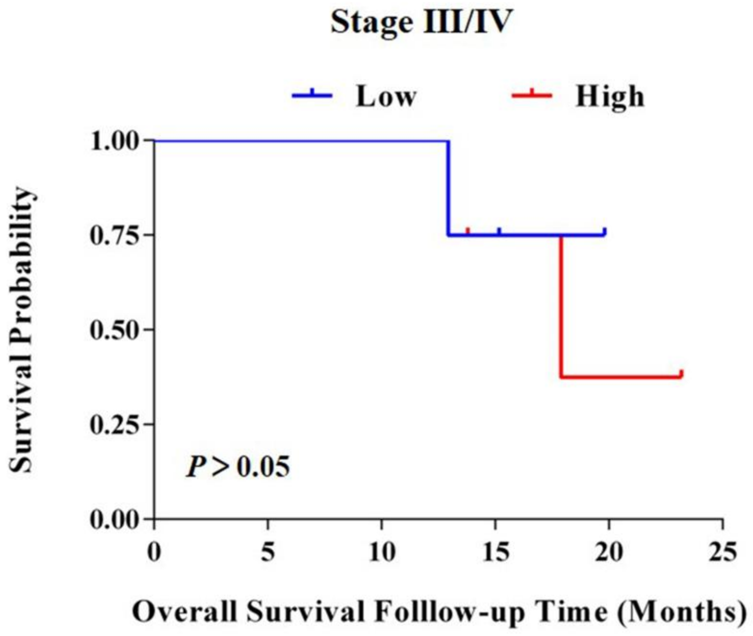

Supplement Figure 1. The prognostic significance of ERCC3 in 8 patients with Stage III/IV pancreatic cancer based on TCGA data mining.
